# Biodiversity-Driven Natural Products and Bioactive Metabolites

**DOI:** 10.3390/plants15010104

**Published:** 2025-12-29

**Authors:** Giancarlo Angeles Flores, Gaia Cusumano, Roberto Venanzoni, Paola Angelini

**Affiliations:** 1 Department of Chemistry, Biology and Biotechnology, University of Perugia, Via del Giochetto, 06122 Perugia, Italy; giancarlo.angelesflores@unipg.it (G.A.F.); gaia.cusumano@dottorandi.unipg.it (G.C.); roberto.venanzoni@unipg.it (R.V.); 2 Centro di Ricerca per l’Innovazione, Digitalizzazione, Valorizzazione e Fruizione del Patrimonio Culturale e Ambientale (CE.D.I.PA.), Piazza San Gabriele dell’Addolorata, 4, 06049 Spoleto, Italy

**Keywords:** biodiversity, natural products, secondary metabolites, plants, fungi, endophytes, marine natural products, metabolomics, biosynthetic gene clusters, synthetic biology, drug discovery

## Abstract

Natural products represent one of the most diverse and functionally sophisticated groups of bioactive molecules found across plants, fungi, bacteria, and marine organisms. Recent advances in genomics, metabolomics, and chemical ecology have fundamentally redefined how these compounds are generated, regulated, and functionally deployed in nature. Increasing evidence reveals that chemical diversity arises not solely from taxonomic lineage but from ecological pressures, evolutionary innovation, and multi-organism interactions that shape biosynthetic pathways over time. Hybrid metabolic architectures, context-dependent activation of biosynthetic gene clusters, and cross-kingdom metabolic integration collectively portray a biosynthetic landscape far more dynamic and interconnected than previously understood. At the same time, mechanistic studies demonstrate that natural products rarely act through single-target interactions. Instead, they influence redox dynamics, membrane architecture, chromatin accessibility, and intracellular signaling in distributed and synergistic ways that reflect both ecological function and evolutionary design. This review synthesizes emerging insights into the evolutionary drivers, ecological determinants, and mechanistic foundations of natural product diversity, highlighting the central role of silent biosynthetic gene clusters, meta-organismal chemistry, and network-level modes of action. By integrating these perspectives, we outline a conceptual and methodological framework capable of unlocking the vast biosynthetic potential that remains dormant within natural systems. Collectively, these advances reposition natural product research as a deeply integrative discipline at the intersection of molecular biology, ecology, evolution, and chemical innovation.

## 1. Introduction

Natural biodiversity is one of the primary drivers of chemical innovation in both terrestrial and marine ecosystems. For millennia, plants, fungi, and marine organisms have represented foundational sources of therapeutic agents, many of which originated in traditional medicinal practices and later became cornerstones of modern pharmacology. Classic examples such as morphine, quinine, digitoxin, and artemisinin illustrate how ecological pressures, including herbivory, microbial antagonism, symbiosis, and environmental stress, shape secondary metabolism and generate compounds with potent biological activities [1,2]. Over the past decades, natural-product research has undergone a profound transformation. Advances in extraction technologies, including ultrasound-assisted, microwave-assisted, and supercritical fluid extraction, together with the widespread adoption of high-resolution analytical platforms such as LC–MS/MS, HRMS, and NMR, have improved dereplication workflows and accelerated structural elucidation [3,4]. These innovations have shifted the discovery pipeline from traditional empirical screening toward data-driven strategies rooted in metabolomics, chemoinformatics, and systems biology. Ethnopharmacology continues to provide valuable insights into plant and fungal chemodiversity, particularly in species historically regarded as toxic. Plants such as Conium maculatum, Nerium oleander, and Strychnos nux-vomica contain potent defensive metabolites that played roles in warfare, hunting, and ritual contexts [5,6,7]. Although these compounds pose clear risks, they have inspired important therapeutic innovations and highlight the thin boundary between toxicity and pharmacological utility [8]. Cultural practices across continents reveal a long-standing empirical understanding of the biological activities embedded in natural metabolomes. Fungal biodiversity represents another expansive reservoir of bioactive metabolites. Endophytic fungi residing within plant tissues produce structurally diverse compounds with antimicrobial, antiviral, anticancer, and immunomodulatory properties [9]. Their intimate association with plants shapes unique biochemical dialogs that modulate biosynthetic pathways. The discovery of taxol-related metabolites in *Taxomyces andreanae* catalyzed worldwide interest in endophyte-derived natural products, even though later genomic studies refined our interpretation of their biosynthetic capacities [10,11]. Today, genome mining, multi-omics integration, and machine-learning tools facilitate the identification and activation of cryptic biosynthetic gene clusters, revealing extensive reservoirs of previously inaccessible fungal chemistry. Marine biodiversity has likewise gained prominence as a source of structurally unconventional metabolites. Organisms adapted to extreme environments—such as those characterized by high pressure, salinity, low light, or specialized symbiotic niches—frequently produce scaffolds with unique oxidation patterns or unusual halogenation. Several marine-derived compounds have already entered clinical use, underscoring the translational potential of chemically specialized marine taxa [12]. Taken together, these insights demonstrate how biodiversity shapes global chemodiversity and supports the continuous emergence of structurally unique metabolites with ecological and pharmacological significance. At the same time, significant challenges persist, including sustainable sourcing, the activation of silent biosynthetic pathways, and the translation of basic discovery into real-world applications. Addressing these challenges requires integrated approaches that combine metabolomics, genome mining, synthetic biology, ecology, and computational tools to unlock the full chemical potential of plants, fungi, and marine organisms.

## 2. Materials and Methods

This scoping review was conducted in accordance with the Preferred Reporting Items for Systematic Reviews and Meta-Analyses extension for Scoping Reviews (PRISMA-ScR) guidelines [13]. The methodological framework was designed to systematically identify, categorize, and synthesize scientific literature concerning biodiversity-derived natural products from plants, fungi, and marine organisms, with a specific emphasis on chemodiversity, biosynthetic mechanisms, and translational applications.

### 2.1. Search Strategy

A comprehensive literature search was performed across four major scientific databases—PubMed, Scopus, Web of Science, and Google Scholar—for the period spanning 2017 to 2026. Only articles published in English and appearing in peer-reviewed journals were considered eligible for inclusion. The search strategy combined controlled vocabulary terms, including Medical Subject Headings (MeSH), with free-text keywords related to natural products, biodiversity, metabolomics, biosynthetic pathways, and biological activity. Boolean operators (AND/OR) were used to structure the queries and refine retrieval. Representative terms included “biodiversity”, “natural products”, “secondary metabolites”, “plant-derived”, “fungal metabolites”, “endophytes”, “marine natural products”, “biosynthetic gene clusters”, “metabolomics”, and “synthetic biology”. To maximize sensitivity across platforms, search strings were adapted to the specific indexing systems and query requirements of each database.

### 2.2. Inclusion and Exclusion Criteria

Studies were included when they focused on natural products or secondary metabolites produced by plants, fungi, endophytes, or marine organisms; when they provided chemical, biological, or ecological characterization of the metabolites of interest; when they described biosynthetic pathways, metabolomic analyses, or biotechnological applications; and when they consisted of original research articles or review papers offering substantive scientific content. These criteria were established to ensure the inclusion of studies capable of contributing meaningfully to a cross-kingdom synthesis of chemodiversity and biosynthesis.

### 2.3. Screening and Selection Process

All retrieved records were imported into a reference management system, and duplicate entries were removed. Following de-duplication, 1842 records remained available for screening. Titles and abstracts were examined to determine their alignment with the inclusion criteria, resulting in the exclusion of 1276 records that clearly did not address the scope of the review. The remaining 566 studies underwent full-text assessment. Articles lacking chemical characterization, offering insufficient reference to biodiversity, or failing to provide substantive analytical or biosynthetic information were excluded. At the end of this process, 180 studies met the eligibility requirements and were incorporated into the final synthesis. Reasons for exclusion were documented at each stage to maintain full methodological transparency.

### 2.4. Data Extraction and Synthesis

Data extracted from each included study addressed the organism of origin, including whether the metabolites were derived from plants, fungi, endophytes, or marine species; the class of metabolites and their structural features; the analytical techniques employed, such as LC–MS/MS, NMR, or other metabolomics workflows; the biosynthetic pathways involved and, when available, the specific biosynthetic gene clusters identified; the ecological context associated with metabolite production, including symbiosis, antagonism, or environmental stress; and the reported biological activities, such as antioxidant, antimicrobial, anticancer, or immunomodulatory effects. Extracted information was synthesized narratively and organized thematically to highlight both convergences and divergences among biological kingdoms. To contextualize emerging scientific directions, additional integrative frameworks—such as metabolomics-guided discovery, genome mining strategies, and One Health perspectives—were incorporated into the analysis.

### 2.5. PRISMA-ScR Flowchart Construction

**Figure 1:**
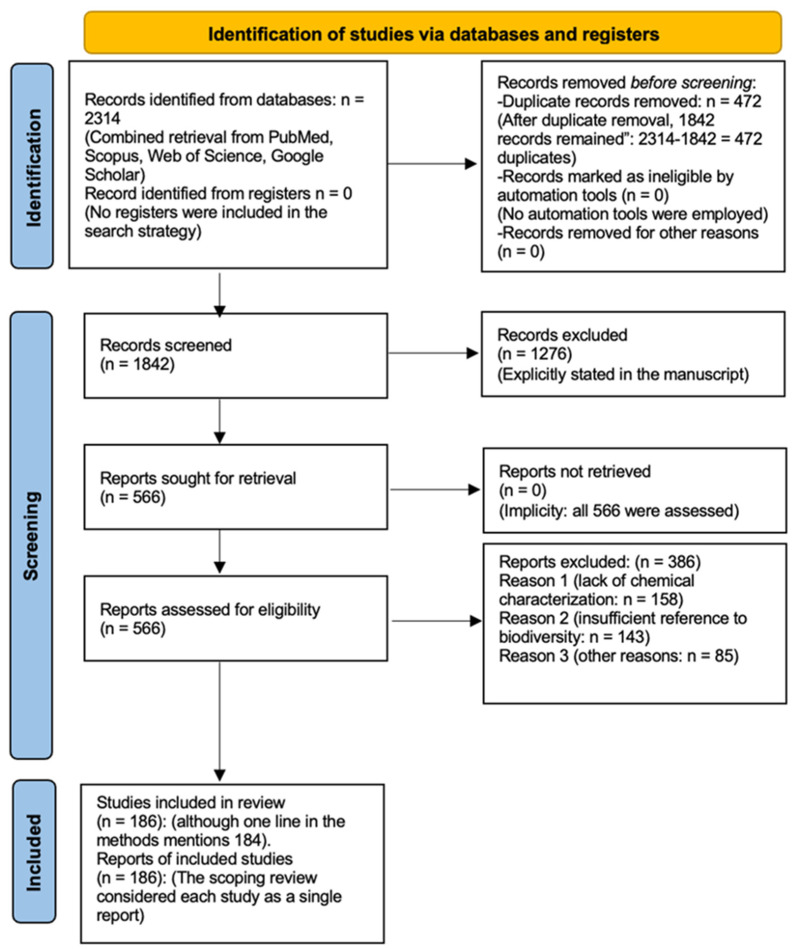
Study flowchart prepared in accordance with the *PRISMA Extension for Scoping Reviews (PRISMA-ScR)* guidelines [13]. The diagram depicts the sequential stages of study identification, screening, eligibility assessment, and final inclusion. It provides a comprehensive overview of the number of records retrieved, excluded, and ultimately incorporated into the review.

A PRISMA-ScR flow diagram was developed to summarize the literature screening process. The flowchart documents the number of records identified (n = 2314), screened after deduplication (n = 1842), assessed for eligibility (n = 566), and ultimately included in the final review (n = 184), together with the primary reasons for exclusion at each step (Figure 1). This visual representation provides an overview of the sequential stages of study identification, screening, eligibility assessment, and inclusion.

### 2.6. Ethical Considerations

As the review is based exclusively on previously published material, no ethical approval was required. All sources were cited appropriately, and data handling followed established academic integrity standards and open-science principles.

## 3. Biodiversity and Its Impact on Chemical Diversity

Biological diversity represents one of the principal forces shaping natural chemodiversity. Plants, fungi, and marine organisms generate structurally complex metabolites whose diversity is molded by evolutionary pressures such as competition, symbiosis, herbivory, and environmental stress. Rather than acting as fixed reservoirs of chemical compounds, these organisms continuously remodel their metabolomes in response to ecological interactions, microhabitat specialization, and environmental cues. The advent of modern metabolomics and genome-mining approaches has demonstrated that biodiversity expands accessible chemical space far beyond what can be achieved through synthetic chemistry or monoculture-based investigations [14,15,16,17].

### 3.1. Fungal Contributions and Interaction-Driven Metabolomes

**Figure 2:**
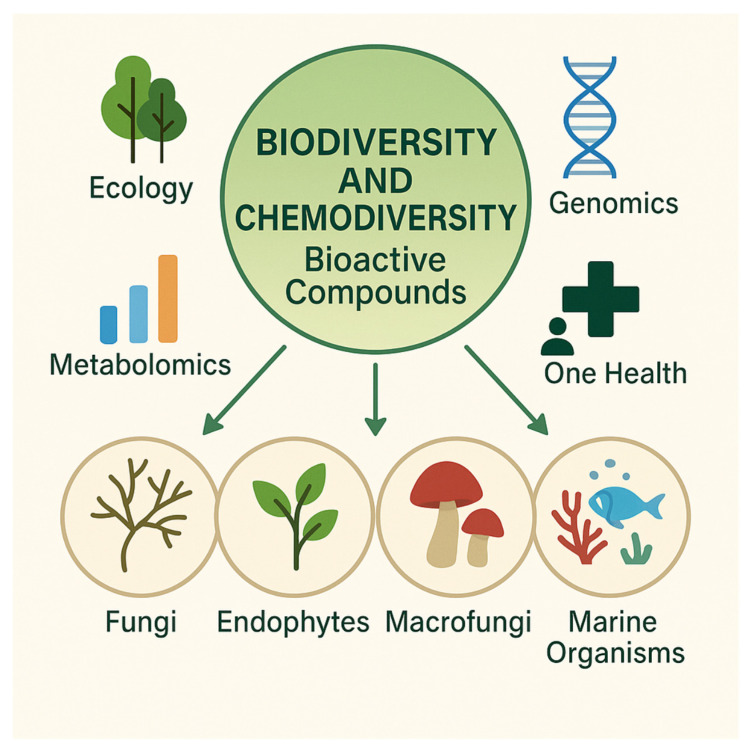
Overview of biodiversity and chemodiversity as sources of bioactive compounds, illustrating major biological groups (fungi, endophytes, macrofungi, and marine organisms) and key conceptual frameworks (ecology, genomics, metabolomics, and One Health) relevant to drug discovery.

Fungi are among the most prolific producers of secondary metabolites, synthesizing polyketides, terpenoids, alkaloids, peptides, and hybrid PKS–NRPS compounds. Their metabolic versatility is closely linked to interspecies interactions, substrate specialization, and ecological adaptability, all of which contribute to the remarkable breadth of fungal chemodiversity [18,19,20]. Endophytic fungi exemplify this dynamic capacity. Living within plant tissues, they engage in constant biochemical exchange with their hosts, triggering lineage-specific and host-modulated biosynthetic pathways. Seminal discoveries such as the detection of taxol-associated metabolites in *Taxomyces andreanae* stimulated global interest in endophyte-derived natural products, although subsequent genomic analyses have clarified the limits and specificities of their biosynthetic capabilities [21,22,23]. Co-culture studies have further demonstrated that ecological interactions markedly expand fungal chemical output. When fungi confront competing organisms—either fungal or bacterial—otherwise silent biosynthetic gene clusters are activated, leading to increases in metabolite diversity of up to fourteen-fold [24,25,26]. These interaction-specific metabolic responses yield metabolites absent in monoculture, including novel cytochalasins, griseofulvin analogs, volatile organic compounds, and polyketides associated with conflict zones. Marine-derived fungi add yet another dimension to fungal chemodiversity. Species associated with macroalgae or inhabiting extreme environments produce halogenated, sulfur-rich, or highly oxygenated scaffolds that reflect adaptation to salinity, oxidative stress, or highly competitive microbiomes [27]. Collectively, such findings demonstrate that fungal chemical diversity is regulated not only by genomic potential but also by ecological stimuli (Figure 2), which act as biotechnological triggers capable of unlocking cryptic biosynthetic pathways [28].

### 3.2. Mushrooms and Mycelial Resources

Macrofungi, including both edible and medicinal mushrooms, make a substantial contribution to natural chemodiversity through the production of terpenoids, phenolic compounds, peptides, and immunomodulatory polysaccharides [29,30]. Their fruiting bodies and mycelial networks generate metabolites associated with lignocellulose degradation, microbial antagonism, protection against UV radiation, and nutrient recycling, reflecting their central role in ecosystem functioning [31]. Recent genomic investigations have revealed that a large proportion of biosynthetic gene clusters in mushrooms remains uncharacterized, indicating a considerable reservoir of hidden metabolic potential [32,33]. Among the best-studied metabolites, β-glucans and other fungal polysaccharides exhibit immunomodulatory, antitumor, and antioxidant properties that can be directly linked to the ecological strategies and adaptive responses of these fungi [34]. Mycelial biotechnology has emerged as a sustainable platform for producing fungal metabolites through controlled fermentation, optimization of growth substrates, and the strategic use of co-culture approaches. Such systems allow high-yield, season-independent production of valuable natural products and underpin the growing industrial interest in mushroom-based and mycelium-based bioprocesses [35,36].

### 3.3. Biodiversity-Informed Drug Discovery

The natural-products discovery pipeline is increasingly shaped by ecological and genomic perspectives, moving beyond broad empirical screening toward approaches that predict which organisms and habitats are most likely to yield structurally novel and biologically relevant metabolites [37,38,39]. Ecological functions such as chemical defense, oxidative stress management, or symbiosis often mirror the pharmacological activities sought in therapeutic development, thereby offering a rational foundation for prioritizing taxa and environmental niches of high bioprospecting potential [40,41]. Genome mining has become a cornerstone of this paradigm, revealing both silent and lineage-specific biosynthetic gene clusters and demonstrating the remarkable abundance of cryptic metabolites in endophytes, symbiotic fungi, and organisms occupying chemically extreme environments [42,43]. Marine ecosystems represent a particularly rich source of biochemical innovation. Selective pressures associated with fluctuating salinity, high hydrostatic pressure, and intimate host–microbe interactions drive the formation of structurally unconventional metabolites that frequently exhibit unique mechanisms of action [44,45,46]. Conceptual frameworks such as One Health reinforce the notion that safeguarding ecosystem integrity is inseparable from protecting the chemical architectures that underpin future therapeutic discovery, antimicrobial innovation, and agricultural resilience [47,48,49]. In this context, chemical biodiversity is recognized not only as a scientific resource but also as a critical component of planetary health.

### 3.4. Towards Sustainable Exploitation of Chemical Biodiversity

Transforming chemical biodiversity into sustainable innovation requires a balance between scientific opportunity and ecological responsibility. Overharvesting and habitat degradation threaten the very ecosystems that generate natural chemodiversity, while alternative strategies—including microbial fermentation, endophyte-based metabolite production, synthetic biology, and heterologous expression—offer more environmentally sustainable routes for accessing valuable natural products [50,51,52]. Global biodiversity governance frameworks, such as the Convention on Biological Diversity, the Nagoya Protocol, and emerging regulations concerning Digital Sequence Information, are redefining access to genetic resources and emphasizing the importance of equitable benefit-sharing [53,54]. The incorporation of conservation biology into chemical, genomic, and biotechnological research is therefore essential to ensure that biodiversity remains a renewable foundation for pharmaceutical, agricultural, and industrial innovation.

## 4. Natural Products Across Biological Kingdoms

Natural products represent one of the most remarkable manifestations of biological innovation, arising from the interplay between evolutionary history, ecological function, and environmental adaptation. Although plants, fungi, and marine organisms differ substantially in physiology, life strategies, and ecological niches, all three groups generate structurally diverse metabolites shaped by selective pressures. These parallels and distinctions form a broad comparative framework for understanding the origins and functional significance of chemodiversity across biological kingdoms [55,56,57]. Plants produce an extensive range of secondary metabolites—including terpenoids, polyphenols, alkaloids, and glycosides—primarily in response to interactions with herbivores, pathogens, and abiotic stress factors. Their sessile nature has driven the evolution of sophisticated chemical defenses and highly specialized signaling molecules that regulate communication with pollinators, symbiotic partners, and the surrounding microbial community. These compounds not only mediate ecological interactions but also contribute to plant resilience under fluctuating environmental conditions [58]. Fungi, by contrast, exhibit a level of metabolic plasticity that reflects their saprotrophic, parasitic, and symbiotic lifestyles. Their ability to colonize heterogeneous substrates, compete with microorganisms, and form intimate associations with plants has led to the diversification of polyketides, terpenoids, nitrogen-containing metabolites, and hybrid PKS–NRPS compounds. The modular organization of fungal biosynthetic pathways frequently results in chemical architectures that are absent in plants and animals, underscoring the evolutionary versatility of fungal metabolism [59]. Marine organisms—including algae, invertebrates, and their associated microbiomes—generate structurally distinctive metabolites shaped by environmental drivers such as high salinity, hydrostatic pressure, intense UV exposure, and complex microbial symbioses. Marine chemodiversity is characterized by unusual halogenation patterns, rare functional groups, and highly oxidized scaffolds, features that reflect adaptation to chemically and ecologically extreme habitats. Such metabolites demonstrate the unique chemical space accessible in marine ecosystems, much of which remains underexplored. A cross-kingdom comparison reveals both divergence and convergence in chemical strategies [60]. Plants often rely on polyphenolic and terpenoid pathways for defense and communication; fungi leverage modular, enzyme-driven biosynthesis to construct diverse polyketides and hybrid metabolites; marine organisms integrate biochemical innovations shaped by extreme environmental pressures and intricate microbial partnerships. Despite their differences, these systems collectively demonstrate that ecological function serves as a powerful driver of structural diversity and bioactivity, reinforcing the central role of evolutionary and ecological dynamics in the generation of natural products [61].

## 5. Chemical Diversity in Natural Products: Evolutionary Drivers, Ecological Pressures and Cross-Kingdom Convergence

Natural products represent one of the most elaborate outcomes of biological evolution. Although they are traditionally organized into discrete chemical families—terpenoids, polyphenols, alkaloids, polyketides, and non-ribosomal peptides—accumulating evidence from comparative genomics, metabolomics, and evolutionary biology increasingly shows that these categories are permeable, interconnected, and shaped by ancient ecological pressures rather than strict taxonomic boundaries [62]. Organisms as distantly related as flowering plants, endophytic fungi, marine macroalgae, filamentous bacteria, and deep-sea invertebrates often produce metabolites that are structurally analogous or functionally convergent, not because of shared ancestry, but because they face similar environmental constraints or selective pressures [63].

The deepest conceptual shift in the last decade concerns the recognition that chemical classes are not static or phylogenetically isolated entities. Instead, they represent dynamic biosynthetic continua that evolve in response to the ecological landscapes in which organisms exist. Interactions such as herbivory, microbial antagonism, symbiosis, nutrient limitation, oxidative stress, or exposure to extreme abiotic conditions exert a profound influence on the architecture and activation of biosynthetic gene clusters (BGCs) [64,65,66]. Parallel evolution, horizontal gene transfer events, and metabolic hybridization further complicate classical definitions of chemical classes, revealing a far greater level of biochemical plasticity than previously recognized [67].

Plants and fungi provide some of the clearest examples of this conceptual reframing. Although they diverged more than a billion years ago, many of their specialized metabolites share overlapping ecological functions, such as deterrence against herbivores, suppression of competing microorganisms, or modulation of redox signals in stress responses [68,69,70,71]. Marine organisms add a further layer of complexity: sponges, algae, and tunicates, living in chemically crowded ecosystems, have evolved metabolic systems characterized by extensive halogenation, hybrid enzymatic architectures, and the incorporation of unusual tailoring reactions, making them particularly rich sources of unprecedented chemical scaffolds [72].

Consequently, understanding chemical diversity in natural products increasingly requires an integrated perspective, one that merges ecological function, evolutionary history, metabolic regulation, and genomic architecture. This section synthesizes recent advances with this integrated lens, emphasizing the evolutionary logic, ecological determinants, and biosynthetic innovations that collectively shape the natural product landscape across biological kingdoms [Table 1].

### 5.1. Evolutionary and Ecological Drivers of Chemical Divergence Across Kingdoms

A central realization emerging from recent research is that the chemical diversity observed across plants, fungi, endophytes, and marine organisms is rooted in convergent ecological demands rather than in shared phylogenetic lineage. Organisms that experience analogous selective pressures tend to evolve chemically related solutions, even when separated by vast evolutionary distances. For example, several plants confronted with herbivore pressure synthesize monoterpenes, sesquiterpenes, and diterpenoids with antifeedant or deterrent activities, a strategy mirrored in numerous pathogenic fungi that deploy structurally similar terpenoids to suppress competing microbes or to modulate plant immune systems during infection [72,73]. The ecological logic is parallel, even if the enzymatic machinery differs. Environmental stressors act as powerful drivers of chemical divergence. In marine macroalgae, for example, repeated exposure to intense UV radiation and osmotic imbalance has selected for brominated and sulfated phenolic compounds with pronounced antioxidant and antimicrobial properties [74]. Plants subjected to drought, salinity, or nutrient limitation respond through coordinated reprogramming of flavonoids, phenylpropanoids, and terpenoids, while fungi exposed to oxidative bursts or bacterial competitors activate polyketide gene clusters that remain silent under benign laboratory conditions [75]. At the genomic level, these ecological pressures shape BGC evolution in multiple ways. In fungi and actinomycetes, BGCs frequently cluster in genomic regions characterized by high recombination rates, facilitating rapid structural diversification under changing environmental conditions [76]. Transposable elements often flank these clusters, creating opportunities for duplication, rearrangement, and recruitment of foreign domains. Horizontal gene transfer events between bacteria and fungi—particularly in soil or marine microbiomes where cell–cell contact is frequent—contribute further to the mosaic nature of specialized metabolism [77]. This phenomenon is especially evident in endophyte-rich plant ecosystems, where close physical and biochemical intimacy allows for metabolic integration between partners. Endophytes may not synthesize the same metabolites as their plant hosts, but they can modulate precursor availability, signaling molecules, and transcriptional regulators, thereby reshaping the plant’s chemical profile. These evolutionary and ecological processes generate a recurring pattern: chemical innovation tends to originate at the interfaces where organisms interact intensely—in rhizospheres rich with microbial competition, on plant surfaces exposed to herbivory, in marine environments subject to constant osmotic stress, or in symbiotic tissues where metabolic networks overlap [78]. Under such conditions, BGCs evolve not as isolated traits but as components of dynamic ecological strategies. One of the most striking consequences of this pattern is the recurrent emergence of structurally analogous metabolites across biological kingdoms. Polyphenols that modulate redox homeostasis in plants show functional convergence with phenolic compounds in marine algae; nitrogen-containing alkaloids deployed as neurotoxins or antifeedants in plants have counterparts in fungal and marine taxa; and terpenoid diversification in fungi displays trends that mirror those observed in plant diterpene evolution [79]. These parallels highlight the extent to which ecological constraints mold biosynthetic potential, repeatedly steering unrelated lineages toward comparable chemical outcomes. Thus, the divergence of natural product classes across kingdoms is not random but follows a coherent evolutionary logic—one that reflects the shared challenges organisms face and the biochemical strategies evolution invents to meet them.

### 5.2. Integrating Genomics and Metabolomics to Redefine Chemical Classes

The integration of high-resolution metabolomics with comparative and functional genomics has radically transformed how chemical classes are delineated and understood. Traditionally, terpenoids, polyketides, alkaloids, or polyphenols were classified according to dominant structural motifs or biosynthetic origins. However, with the advent of genome mining, gene-cluster mapping, and metabolite–gene correlation analyses, these distinctions increasingly appear porous. Evidence from multiple biological kingdoms demonstrates that the biosynthetic architecture underlying these compounds is far more interconnected—and evolutionarily fluid—than previously assumed. One of the clearest illustrations of this conceptual shift is the expanding recognition of hybrid biosynthetic architectures [80]. In fungi, particularly in genera such as *Aspergillus* and *Penicillium*, terpene synthases frequently co-occur with polyketide synthase (PKS) modules, giving rise to meroterpenoids that fuse isoprenoid skeletons with highly oxygenated polyketide moieties. These hybrid scaffolds challenge the conventional separation between terpenoid and polyketide pathways, revealing instead a shared enzymatic landscape shaped by co-option, duplication, and domain fusion events. In marine organisms, similar trends have emerged: halogenated meroterpenoids and PKS–terpene hybrids isolated from macroalgae and sponges exhibit biosynthetic signatures consistent with modular enzymatic crosstalk rather than isolated routes [81]. Such findings suggest that hybridization is not an exception but rather a recurring outcome in ecologically dynamic environments. Metabolomics has been critical to unmasking these relationships. Untargeted LC–MS and NMR-based profiling consistently show that compounds historically assigned to distinct chemical classes share precursor pools or co-regulated metabolic fluxes under specific environmental conditions. For instance, stress-induced phenylpropanoid accumulation in plants often correlates with the upregulation of terpenoid and flavonoid pathways, hinting at shared transcriptional regulators and cross-pathway metabolic dialog [82]. In fungal systems, the expression of multiple BGCs—some encoding terpenes, others polyketides or hybrid scaffolds—can be synchronized in response to bacterial competitors or plant-derived signaling molecules, a phenomenon that becomes visible only when genomic data are analyzed alongside metabolomic time-series datasets [83]. Such integrative analyses have also revealed the vast breadth of silent biosynthetic potential across fungi, plants, and marine organisms. Genome mining studies frequently show that detectable metabolites represent only a fraction of the underlying metabolic repertoire: in fungi, for example, the majority of PKS and NRPS gene clusters remain unexpressed under standard laboratory conditions [84]. Metabolomic profiling under co-culture or stress exposure, however, routinely unveils compound families that cannot be traced back to known pathways, implying the activation of cryptic or hybridized biosynthetic systems that were previously unrecognized. Another major advancement derived from integrative omics is the identification of coevolutionary signatures between hosts and their associated microbiota. Endophytes living within plant tissues modulate host gene expression, metabolic fluxes, and precursor availability, producing emergent metabolite profiles that differ markedly from either partner in isolation [85]. Studies in *Taxus*, *Curcuma*, *Artemisia*, and other medicinal plants illustrate that plant–microbe consortia form metabolic continua in which genes, signals, and precursors circulate bidirectionally. The result is an expanded chemical landscape where host-derived and microbe-derived pathways overlap, merge, or mutually modulate each other’s outputs. In marine ecosystems, integrative genomics has uncovered a similar pattern: sponges and tunicates frequently host dense microbial consortia whose biosynthetic genes account for the majority of structurally complex metabolites historically attributed to the animal host [86,87]. Metabolomics-guided genomic reconstruction has shown that many “marine natural products” arise from intricate multi-organism networks, with host tissues providing structural scaffolding, while associated bacteria or fungi supply the biosynthetic machinery. Taken together, genomics and metabolomics converge toward a unified conclusion: chemical classes, as historically defined, fail to capture the evolutionary and ecological complexity of natural product biosynthesis. Pathways once thought to be independent are part of interconnected regulatory and metabolic frameworks that respond collectively to ecological pressures. This realization not only enriches our understanding of chemical diversity but also provides a predictive foundation for future discovery—by identifying ecological contexts and genomic configurations that are most likely to yield novel metabolites.

### 5.3. Breakthroughs in Major Chemical Families (2020–2025)

The past five years have been marked by particularly rapid progress in the characterization and understanding of natural product families. These developments do not merely expand the catalog of known molecules but challenge long-standing assumptions about biosynthetic logic, ecological function, and evolutionary history. Although the classical families—terpenoids, polyphenols, alkaloids, polyketides, and NRPs—remain useful conceptual anchors, their boundaries appear increasingly permeable, and the contextual determinants of their biosynthesis are more influential than the structural categories themselves [88,89].

**Terpenoids**

Terpenoids continue to represent one of the most chemically and functionally versatile classes, but the emergence of hybrid PKS–terpene pathways has dramatically reshaped our understanding of their biosynthetic origins. Genomic surveys of species such as *Aspergillus*, *Penicillium*, and several *Clavicipitaceae* taxa have revealed terpene synthase genes colocalizing with polyketide modules or oxidative tailoring enzymes, resulting in meroterpenoids whose architectures deviate significantly from canonical cyclization patterns [90]. These compounds often display potent antimicrobial or antifeedant properties, indicating their ecological functions extend beyond primary defense to include communication, competitive exclusion, and even redox signaling. Marine organisms contribute additional layers of novelty. Macroalgae, sponges, and marine-derived fungi synthesize halogenated terpenoids with oxidation patterns rarely observed in terrestrial systems. Such metabolites frequently possess antiviral or cytotoxic properties, and their biosynthetic routes involve halogenases, desaturases, and P450 enzymes adapted to high-salinity or high-oxidative-pressure environments [91,92,93,94]. These biochemical adaptations underscore the capacity of terpenoid pathways to reconfigure themselves in response to extreme ecological contexts.

**Polyphenols**

Polyphenols, historically regarded as antioxidants, have undergone a conceptual re-evaluation as research increasingly highlights their roles as modulators of redox-responsive signaling pathways. Plants experiencing abiotic stresses such as drought, salinity, or UV exposure undergo deep reprogramming of the phenylpropanoid and flavonoid pathways, yielding metabolites that influence Nrf2- and MAPK-mediated responses [95].

Metabolomics integrated with microbial ecology reveals that these responses are rarely autonomous. Endophytic fungi and bacteria modify plant polyphenolic profiles by modulating precursor availability or by producing enzymatic regulators that redirect metabolic fluxes. This interdependence suggests that many polyphenols should be reinterpreted not as isolated plant metabolites, but as emergent products of meta-organismal chemistry, reflecting the combined biosynthetic capacities of hosts and their microbiota [96]. In marine systems, polyphenols take on different ecological roles. Brown algae produce brominated phlorotannins that function as potent antibiofilm and antiviral agents, shaping their interactions with epiphytic microorganisms and grazers. These metabolites illustrate how ecological pressures specific to marine habitats—such as oxidation gradients, microbial colonization, and salinity—drive the emergence of structurally unusual phenolic scaffolds [97,98].

**Alkaloids**

Alkaloids remain among the most pharmacologically potent natural product classes, but recent genomic studies have corrected several persistent misconceptions. Among the most notable is the case of *Taxomyces andreanae* and taxol production. Early analytical studies suggested that certain endophytes could independently synthesize taxol, but comprehensive genomic analyses unequivocally showed the absence of a complete BGC in *Taxomyces*, revealing that earlier observations reflected analytical artifacts rather than true biosynthesis [99].

Despite this clarification, alkaloid innovation continues at a rapid pace. Fungi produce highly oxygenated indole diterpenoids with neuroactive and antifeedant properties, and marine invertebrates yield halogenated alkaloids with strong antiviral or cytotoxic effects [100,101]. These compounds often arise via hybrid pathways, incorporating NRPS modules or terpenoid precursors, and their ecological functions reflect their structural diversity: from modulating insect behavior to suppressing microbial competitors.

**Polyketides**

Polyketides constitute the most structurally diverse natural product family, but their biosynthetic potential far exceeds the compounds traditionally isolated. Genome mining has revealed an overwhelming prevalence of silent or poorly expressed PKS clusters, particularly in fungal taxa [102]. Advances in epigenetic remodeling, including the use of histone deacetylase inhibitors or DNA methyltransferase inhibitors, have enabled the activation of some of these clusters, leading to the discovery of new cytotoxic or antimicrobial molecules. CRISPR-based transcriptional activation has provided a more targeted approach, although its success depends on the integrity of regulatory networks that are often only partially understood. Ecologically inspired co-culture experiments have delivered some of the most striking results. When fungi are grown alongside bacteria or other fungi, gene expression patterns shift dramatically, activating BGCs that remain silent in monoculture and producing polyketides with entirely new scaffolds or biological properties [103]. These findings underscore the principle that polyketide biosynthesis is deeply responsive to ecological stimuli.

**NRPs and PKS–NRPS hybrids: frontier chemistry**

NRPs and their hybrid counterparts represent the frontier of biosynthetic innovation. These molecules routinely incorporate non-proteinogenic amino acids, unusual heterocycles, or extended polyketide segments, yielding architectures that defy classical classifications [104]. Many NRPs serve as mediators of microbial competition, quorum-sensing interference, or symbiotic communication. Recent work on plant-associated fungal consortia shows that NRPs can help stabilize plant–microbe interactions, modulate host immunity, or inhibit microbial pathogens, revealing their multifaceted ecological roles [105]. Collectively, breakthroughs from 2020 to 2025 reveal that chemical classes are neither isolated nor rigid but form dynamic, ecologically responsive networks shaped by hybrid biosynthetic logic.

### 5.4. Ecological Logic and Cross-Kingdom Convergence

Natural product diversity is often presented as the outcome of biochemical complexity, yet mounting evidence suggests that ecology—not chemistry—provides the primary organizing principle of metabolite evolution. Chemical convergence across kingdoms is most pronounced in environments characterized by intense interaction networks. Plants under herbivore pressure evolve terpenoids and alkaloids almost indistinguishable in function from those produced by fungi defending their substrates from bacterial competitors [106]. Marine macroalgae synthesize brominated phenolics and halogenated terpenoids that mirror the defensive metabolites of marine-derived fungi inhabiting similar ecological niches. These parallels illustrate that analogous selective pressures repeatedly drive unrelated lineages toward comparable chemical strategies. The ecological logic underlying this convergence is multifaceted [107]. Chemical interactions mediate competitive hierarchies in soil and marine microbial communities. In the rhizosphere, for example, root exudates rich in terpenoids and phenolic compounds regulate microbial colonization patterns while simultaneously shaping fungal BGC expression. In turn, microbial metabolites influence plant immune responses and stress signaling, giving rise to bi-directional chemical dialogs that integrate multiple metabolic pathways across different organisms. Symbiotic systems embody this complexity most clearly. In plant–endophyte associations, endophytes do not merely inhabit plant tissues; they actively participate in shaping host chemical profiles by modulating precursor pools, altering transcriptional regulators, or synthesizing signaling molecules that redirect host metabolism [108]. The resulting metabolome is not the sum of two independent chemistries but an emergent system, reflecting coevolutionary compromise and metabolic interdependence. Marine holobionts offer another instructive example. Sponges and tunicates host dense microbial consortia whose biosynthetic repertoires explain the majority of structurally complex “animal-derived” metabolites historically attributed to the hosts themselves [109]. Here, ecological stability relies on chemical regulation: host tissues provide structural scaffolding, while microbial partners manufacture metabolites that mediate defense, microbial balance, and communication. These examples collectively demonstrate that chemical convergence is not a coincidence but a predictable outcome of ecological and evolutionary constraints. When distinct organisms confront similar challenges—competitive exclusion, oxidative stress, microbial colonization, herbivory—they tend to evolve biosynthetic solutions that, despite originating from different gene clusters or enzymatic lineages, are functionally analogous [110]. From this perspective, natural product diversity becomes intelligible as a set of adaptive strategies distributed across kingdoms, shaped by the recursive interplay of genomic innovation and ecological necessity.

### 5.5. Emerging Themes, Knowledge Gaps, and Conceptual Advances

The synthesis of recent advances across plants, fungi, endophytes, and marine organisms reveals a series of unifying conceptual themes that fundamentally reshape our understanding of natural product biosynthesis. The first major theme is the growing recognition that hybrid biosynthetic logic is far more pervasive than previously assumed. Pathways once believed to operate in isolation—such as terpenoid and polyketide systems, or NRPS and terpene synthase routes—are now known to interconnect through domain fusion, co-regulation, and evolutionary co-option [111]. Meroterpenoids in fungi, halogenated hybrid scaffolds in marine macroalgae, and plant–endophyte metabolic synergies demonstrate that chemical classes frequently merge at both structural and regulatory levels. The classical taxonomies of natural product chemistry, while still useful for description, no longer capture the dynamic and modular nature of these biosynthetic networks [112,113]. A second theme concerns the central role of ecological cues as primary regulators of metabolic expression. Traditional cultivation conditions reveal only a fraction of an organism’s biosynthetic potential, in part because they fail to reproduce the ecological pressures that naturally activate specialized metabolism [114]. Co-culture experiments, plant–microbe signaling systems, and stress-induction assays consistently demonstrate that biosynthetic gene clusters (BGCs) respond to competitive, oxidative, or symbiotic stimuli rather than to laboratory-standard conditions. This ecological dependency explains why many biosynthetic pathways remained undocumented for decades and why recent methodological shifts—particularly the integration of ecological simulation with multi-omics—have yielded such rapid expansions in chemical discovery [115]. The third theme emerging from cross-kingdom analyses is the sheer scale of latent biosynthetic capacity. Genome mining studies reveal that fungi, plants, and marine organisms harbor dozens to hundreds of silent or cryptic BGCs whose products remain unknown [116,117]. These clusters encode unprecedented chemical logic—including novel cyclization mechanisms, unconventional tailoring reactions, and hybrid modular systems—yet their activation requires precise ecological or genetic stimuli. Unlocking this reservoir is arguably the most compelling frontier in natural product research, with broad implications for pharmacology, ecology, and evolutionary biology. Despite these conceptual advances, important knowledge gaps persist. One of the most challenging questions concerns how organisms coordinate multi-pathway responses to complex environmental challenges. Stress-induced metabolite profiles often involve simultaneous modulation of terpenoid, phenylpropanoid, alkaloid, and polyketide pathways, suggesting that higher-order regulatory circuits govern metabolic prioritization under ecological pressure. Yet the identity, hierarchical organization, and evolutionary origins of these circuits remain poorly understood [118].

Another unresolved area involves the ecological triggers that consistently activate cryptic BGCs. While some patterns are emerging—such as bacterial antagonism in fungi, herbivory in plants, or oxidative bursts in marine habitats—the full spectrum of stimuli capable of awakening silent clusters is far from mapped. Furthermore, the evolutionary determinants that favor the emergence of hybrid pathways in certain lineages but not others remain largely speculative. Genomic stability, ecological context, and metabolic architecture all appear to play roles, but their interactions require deeper inquiry [119].

Finally, the increasing recognition of meta-organismal chemistry, particularly in plant–endophyte and host–microbiome systems, raises fundamental questions about the boundaries of biosynthesis. In many associations, metabolites cannot be attributed unambiguously to a single organism, challenging classical assumptions about biosynthetic ownership and suggesting that natural product discovery must increasingly embrace community-level frameworks [120]. Addressing these gaps will be essential for accessing the full biosynthetic potential of natural systems and forms the conceptual bridge to the mechanistic perspectives explored in the following section.

## 6. Mechanisms of Action of Natural Products Across Kingdoms

**Figure 3:**
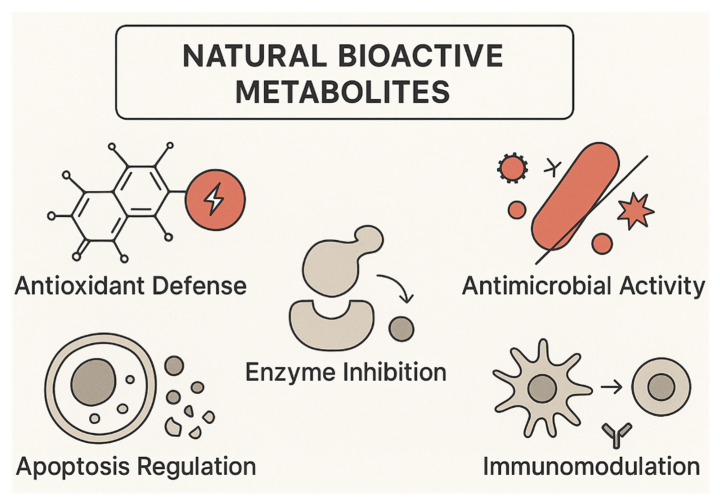
Schematic representation of the principal molecular mechanisms through which natural bioactive metabolites exert their effects. These include antioxidant defense, antimicrobial activity, enzyme inhibition, modulation of apoptosis, and immunomodulatory actions.

Understanding the mechanisms by which natural products exert their biological effects is essential to interpreting their ecological roles, their evolutionary significance, and their potential applications. While classical descriptions typically categorized mechanisms as antioxidant, antimicrobial, anti-inflammatory, or cytotoxic, recent advances in molecular biology and chemical ecology reveal that these labels are insufficient. Natural product activity often emerges from multilayered interactions, combining direct biochemical effects with alterations of signaling networks, transcriptional programs, redox balance, and host–microbe communication (Figure 3). These mechanisms are not merely conserved across kingdoms but often arise through convergent evolution driven by similar ecological pressures [121]. Mechanistic insights have benefited particularly from transcriptomic profiling, receptor-binding studies, metabolite–protein interactomics, and imaging-based approaches that capture the spatial context of compound action. Such analyses show that most natural products act not through a single defined target but rather through distributed regulatory networks, engaging multiple nodes and initiating cascading physiological responses [122].

### 6.1. Redox Modulation as a Regulatory, Not Merely Protective, Mechanism

Redox modulation remains one of the foundational mechanisms underlying the biological activity of many natural products, but the contemporary view extends far beyond the simplistic “antioxidant” paradigm. Polyphenols, terpenoids, and certain alkaloids operate as fine-tuned regulators of redox-sensitive transcriptional circuits, influencing cellular decision-making in plants, fungi, and marine organisms alike. Flavonoids and phenylpropanoids, for example, modulate ROS signaling by altering hydrogen peroxide flux, controlling the activation of MAPK cascades, and influencing transcription factors such as WRKY, MYB, and bZIP families in plants under stress [123,124]. Rather than merely scavenging radicals, these compounds adjust the amplitude and duration of oxidative signals, thereby modulating stress responses, pathogen recognition, and metabolic reprogramming. In marine macroalgae, brominated phenolics act similarly as modulators of oxidative bursts, balancing ROS production during interactions with competing microorganisms or grazers [125]. Fungal phenolic metabolites and polyketides regulate redox homeostasis by influencing mitochondrial respiration and oxidative enzyme activity, often in response to bacterial antagonism. These redox-based mechanisms illustrate a generalizable strategy by which organisms integrate environmental signals into biochemical responses [126].

### 6.2. Membrane Disruption, Ion Homeostasis, and Structural Perturbation

Membrane-targeting mechanisms represent another major axis of natural product action, particularly among terpenoids, polyketides, and alkaloids. Many compounds insert into lipid bilayers due to their amphipathic nature, altering membrane fluidity, permeability, and ion gradients. In fungi, for example, highly oxygenated meroterpenoids integrate into ergosterol-rich membranes, generating localized disruptions that impair proton pumping and ATP synthesis [127]. Marine-derived halogenated terpenoids affect the lipid microdomain organization of grazers or epiphytic bacteria, collapsing electrochemical gradients and inhibiting motility [128]. Plants utilize membrane-active terpenoids and alkaloids as a first line of defense against pathogens: monoterpenes can permeabilize microbial membranes, while alkaloids interfere with membrane-bound enzymes involved in respiration or cell division. Even subtle structural differences—chain branching, halogenation, methylation—may drastically modify membrane affinity and target specificity [129,130]. Through such interactions, membrane perturbation acts not only as a direct antimicrobial strategy but also as a means to modulate signaling and homeostasis in the producing organism itself.

### 6.3. Interference with Essential Enzymes and Biosynthetic Pathways

A large subset of natural products exerts its biological effects by modulating key enzymatic hubs in central or specialized metabolism. Polyketides are particularly known for such mechanisms because their structural complexity allows precise, high-affinity interactions with catalytic pockets. Fungal polyketides often inhibit oxidative enzymes or dehydrogenases, disrupting carbohydrate metabolism or interfering with fungal–bacterial competition dynamics [131,132]. Marine alkaloids commonly target topoisomerases and DNA polymerases, contributing to their observed cytotoxicity [133]. In plants, alkaloids such as benzylisoquinolines act on acetylcholinesterase-like enzymes in herbivores, disturbing neurotransmission and deterring feeding. NRPs and PKS–NRPS hybrids provide some of the most sophisticated examples of enzymatic interference. Many bacterial NRPs inhibit quorum-sensing regulators or carbonic anhydrases, altering microbial behavior, communication, and virulence [134]. Their ecological significance often extends beyond killing competitors: by modulating enzyme networks, they reconfigure community structure and resource allocation.

### 6.4. Disruption of Cellular Signaling Networks Pathways

A unifying theme across kingdoms is that natural products frequently act by rewiring signaling networks rather than by targeting single enzymes. In plants, diterpenoids and sesquiterpenes influence hormone signaling pathways—including jasmonate, salicylate, and ethylene—thereby modulating immune responses, cell death programs, and stress adaptation [135]. Endophyte-associated metabolites further complicate this picture. By altering host hormone levels or mimicking plant signaling molecules, endophytes can reshape transcriptional programs in ways that benefit both partners [136]. These interactions demonstrate that signaling disruption or enhancement is often a cooperative phenomenon, emerging from symbiotic integration rather than antagonism. Marine organisms provide parallel examples: sponge-associated bacteria produce metabolites that modulate host immune pathways, regulating inflammation, cell adhesion, and tissue remodeling [137]. Fungi subjected to microbial antagonism activate complex signaling cascades involving MAPKs, calcium fluxes, and G-protein-coupled pathways, all of which are targeted by stress-induced metabolites [138,139].

### 6.5. Epigenetic Modulation and Activation of Silent Gene Clusters

One of the most exciting areas of mechanistic research involves the epigenetic modulation of biosynthetic gene clusters (BGCs). Fungi and bacteria harbor vast reservoirs of silent or poorly expressed clusters that can be awakened by epigenetic reprogramming, often mediated by natural products or ecological factors. Histone deacetylase inhibitors, for instance, can activate fungal PKS clusters, leading to the emergence of novel polyketides not detectable in untreated cultures [140,141]. Interactions with bacteria or plant hosts may induce changes in chromatin accessibility, transcription factor activity, or noncoding RNA profiles, stimulating the expression of cryptic clusters that encode unprecedented chemical scaffolds [142]. In plants, chromatin remodeling plays a central role in the stress-induced activation of secondary metabolism. DNA methylation changes and histone modifications modulate the transcription of terpenoid synthases, phenylpropanoid enzymes, and alkaloid biosynthetic genes, allowing plants to adjust their metabolic outputs in response to fluctuating ecological pressures [143]. Thus, epigenetic regulation forms a mechanistic bridge between ecological stimuli and biosynthetic innovation, revealing how organisms translate environmental signals into chemical diversity.

### 6.6. Multi-Target and Network-Level Mechanisms

Perhaps the most critical contemporary insight is that natural products typically operate through multi-target mechanisms that cannot be reduced to classical “single-hit” models [144]. Transcriptomic and proteomic studies demonstrate that terpenoids, alkaloids, phenolics, and polyketides simultaneously engage multiple cellular systems—including redox regulation, membrane stability, metabolic flux, transcriptional control, and enzyme inhibition—producing synergistic effects that shape organismal physiology. This network-level perspective explains both the ecological potency and the structural diversity of natural products [145,146]. Compounds with modest affinity for individual targets can exert profound biological impacts when they modulate multiple pathways in concert, particularly in dynamic ecosystems where organisms must integrate numerous environmental signals.

### 6.7. Conceptual Integration

Taken together, mechanistic studies reveal that natural products function not as isolated biochemical agents but as regulatory instruments that organisms employ to navigate ecological challenges. Their activity arises from distributed interactions across membranes, enzymes, chromatin, and signaling networks—interactions that vary according to environmental context and evolutionary history. These mechanistic insights set the stage for the next section, which explores how natural products, ecological dynamics, and biosynthetic potential converge to shape future research directions.

## 7. Results and Thematic Review

Over the past five years, the collective body of research has converged on a central insight: biodiversity is not a static catalog of organisms and metabolites but a dynamic, interaction-driven engine of chemical innovation. Across biological scales—including microbial consortia, endophytes, macrofungi, and marine holobionts—ecological interactions have emerged as decisive modulators of metabolomic architecture. These interactions reshape chemical profiles, activate cryptic biosynthetic gene clusters, and reveal reservoirs of structural diversity far richer than those accessible through monoculture-based approaches. This interaction-dependent metabolic plasticity underscores the pivotal role of fungi and their mycelial networks as both ecological stabilizers and prolific sources of bioactive secondary metabolites with therapeutic, agricultural, and industrial relevance [147,148]. Advances in genome mining, high-resolution metabolomics, and synthetic biology have further illuminated the magnitude of this latent biosynthetic potential. Although thousands of biosynthetic gene clusters (BGCs) have been annotated across plants, fungi, and marine organisms, only a minority are naturally expressed under conventional laboratory conditions. Consequently, current research increasingly focuses on elucidating the regulatory, ecological, and evolutionary principles that maintain BGC silence, despite the sophistication of modern predictive tools [149,150]. Innovative activation strategies—most notably co-culture systems that mimic ecological pressures, epigenetic modulation of chromatin accessibility, and heterologous expression platforms capable of bypassing native regulatory constraints—have proven indispensable for accessing these dormant pathways. Together, such approaches are dramatically expanding the repertoire of discoverable metabolites and enabling the systematic exploration of previously inaccessible chemical space [151,152]. Parallel developments in biodiversity-informed drug discovery continue to highlight the exceptional pharmaceutical value embedded in natural metabolomes. Yet these advances also sharpen the awareness that biodiversity loss equates to the irreversible erosion of molecular possibilities. Species extinction eliminates unique evolutionary solutions that cannot be recreated synthetically, emphasizing that biodiversity represents both an irreplaceable evolutionary legacy and a foundational resource for future biomedical innovation [153,154]. At the interface of scientific discovery and environmental responsibility, emerging frameworks stress that the exploitation of biological resources must proceed in concert with principles of ecological stewardship. International agreements—including the Convention on Biological Diversity and the Nagoya Protocol—provide ethical and legal foundations for equitable benefit-sharing, while green biotechnologies, circular bioeconomy strategies, and the valorization of biological residues exemplify pathways toward sustainable innovation [155,156]. Within this broader context, the integration of fungi into global sustainability strategies is gaining increasing recognition. Their ecological functions—ranging from nutrient cycling to soil regeneration and climate resilience—position them as indispensable allies in the development of sustainable food systems, climate-mitigation approaches, and public-health interventions [157]. Taken together, the emerging evidence paints a cohesive thematic picture: biodiversity is simultaneously heritage and horizon—heritage as the cumulative product of deep evolutionary time, and horizon as a vast, still-unexplored repository of biochemical solutions to medical, environmental, and societal challenges.

## 8. Future Directions and Critical Perspectives in Natural Product Research

The integration of ecological, genomic, and mechanistic perspectives has reshaped the conceptual foundations of natural product research. Yet, the most transformative advances are likely to emerge from approaches that transcend conventional disciplinary boundaries. Future progress will depend not only on discovering new compounds but also on understanding the ecological logic, regulatory architecture, and evolutionary dynamics that generate chemical diversity across kingdoms [158,159]. This section outlines the conceptual trajectories, methodological innovations, and unresolved challenges that will define the next decade of research in natural product chemistry and chemical ecology.

### 8.1. Embracing Ecology as a Predictive Framework for Discovery

The prevailing paradigm in natural product discovery has long relied on taxonomic screening and artificial cultivation conditions. However, as demonstrated by multiple studies in plants, fungi, and marine organisms, biosynthetic expression is fundamentally ecology-dependent [160,161]. Compounds are not produced at random but arise from selective pressures—competition, herbivory, symbiosis, oxidative stress—that shape metabolic architecture over evolutionary time. Future discovery efforts should therefore transition from taxonomy-driven to ecology-driven strategies. Targeted sampling of competitive microbial consortia, extreme environmental niches, marine holobionts, or plant–endophyte symbioses is far more likely to reveal unusual scaffolds than untargeted organismal screening [162,163]. The increasingly recognized phenomenon of chemical convergence across kingdoms reinforces this point: structurally analogous metabolites often emerge in ecologically similar contexts, regardless of phylogeny [164]. Developing ecological models capable of predicting when and where chemical novelty is most likely to occur represents an important frontier, particularly when combined with metabolomic and genomic profiling.

### 8.2. Unlocking Silent Biosynthetic Potential

Perhaps the most compelling challenge for the field lies in the activation of silent or cryptic biosynthetic gene clusters (BGCs). Genome-mining analyses consistently reveal that a substantial portion of biosynthetic capacity remains unexpressed under standard laboratory conditions, with many clusters encoding reactions and scaffolds entirely absent from currently characterized chemical space [165]. Unlocking this hidden metabolic potential will require activation strategies informed by the ecological triggers that naturally regulate these pathways in vivo. Several experimental directions are beginning to shape this effort. Epigenetic remodeling—through histone deacetylase inhibition or targeted modulation of DNA methylation—has shown promise in relieving transcriptional repression and reawakening dormant clusters [166]. Co-culture systems capable of recreating antagonistic or symbiotic interactions likewise induce BGC expression by exposing organisms to the chemical and physical cues that govern metabolic competition in nature [167]. CRISPR-based transcriptional activation offers a more direct route, enabling selective upregulation of specific clusters, while the controlled simulation of herbivory-related signals, bacterial quorum molecules, or oxidative stress mimics complex ecological conditions that frequently elicit specialized metabolism. Despite these advances, a deeper and still unresolved question persists: why do certain clusters remain silent even under multifactorial stimulation? Addressing this problem will require elucidating how regulatory hierarchies, chromatin architecture, and metabolic flux intersect to gatekeep BGC expression [168,169]. Fully understanding this convergence will demand a synthesis of ecological insight, chromatin biology, and systems-level modeling—an integrative perspective essential for accessing the immense biosynthetic potential that remains hidden within natural systems.

### 8.3. Reconstructing Meta-Organismal Chemistry

A growing body of evidence indicates that many natural products are emergent properties of multi-organism systems, particularly in plant–endophyte networks and marine holobionts. Endophytes modulate host metabolism, influence precursor flow, and induce transcriptional responses that fundamentally reshape metabolite profiles [170]. Marine sponges and tunicates host microbial communities whose biosynthetic machinery accounts for a substantial portion of structurally complex metabolites attributed to the animal host [171]. To move forward, natural product chemistry must adopt meta-organismal frameworks in which the metabolic contributions of host, microbiota, and environment are analyzed collectively rather than in isolation. Advanced spatial metabolomics, single-cell transcriptomics, and high-resolution imaging will be essential for resolving the spatial organization and metabolic interdependence of these networks [172].

Conceptually, this shift raises fundamental questions about biosynthetic ownership, evolutionary selection, and the nature of chemical adaptation—questions that will likely redefine how we classify and interpret natural products in the next generation of research.

### 8.4. Mechanistic Integration Across Levels of Biological Organization

Mechanistic studies increasingly reveal that natural products exert their activity not through discrete, single-target interactions but through a distributed, network-level modulation that engages membranes, enzymes, chromatin, and complex signaling circuits simultaneously [173,174]. This multilayered complexity underscores the need for integrative platforms able to connect molecular interactions with pathway dynamics, translate these dynamics into organismal responses, and ultimately interpret their ecological consequences. Advancing this goal will require research strategies that synthesize multiple analytical dimensions. Multi-omics approaches—encompassing transcriptomics, proteomics, metabolomics, and chromatin accessibility—must be combined to capture the full spectrum of biochemical and regulatory changes elicited by natural products [175,176]. High-throughput interactomic techniques capable of mapping distributed molecular targets will be essential for identifying the nodes most affected within these networks. Equally important is the development of dynamic models that describe how natural products reshape signaling pathways over time, as well as comparative mechanistic studies across kingdoms that can reveal whether convergent mechanisms arise from shared evolutionary constraints or reflect repeated biochemical solutions to similar ecological pressures [177]. Together, these integrative frameworks promise to clarify the extent to which mechanisms such as redox modulation or membrane disruption represent universal adaptive strategies, and how they contribute to the remarkable functional diversity observed across natural product chemistry (Table 2).

## 9. Conclusions

Natural products represent one of the most dynamic and conceptually rich arenas of contemporary biological research. The integration of ecological, genomic, and mechanistic perspectives over the past decade has fundamentally rewritten our understanding of how chemical diversity emerges, adapts, and functions across biological kingdoms. Rather than existing as static taxonomic categories, natural product families now appear as fluid, interconnected systems shaped by ecological pressures, regulatory hierarchies, evolutionary innovation, and cross-kingdom interactions [178]. The themes developed throughout this review—biosynthetic plasticity, ecological convergence, mechanistic complexity, and latent metabolic potential—reveal that chemical diversity is best interpreted through an integrative lens [179,180]. Plants, fungi, endophytes, marine organisms, and bacteria generate structurally and functionally analogous compounds not because of shared ancestry, but because they inhabit ecological spaces that impose similar selective demands. This ecological logic, in turn, governs the activation of biosynthetic gene clusters, the organization of metabolic pathways, and the evolution of hybrid or unconventional scaffolds [181]. Mechanistic studies underscore that natural products rarely operate through single-target interactions. Instead, they modulate signaling networks, redox circuits, membrane dynamics, and enzymatic pathways in ways that reflect both the biochemical constraints of the producing organism and the ecological functions the compounds serve [182]. These distributed mechanisms highlight the need for more integrative, multi-scale approaches capable of linking molecular interactions with physiological responses and ecological outcomes. At the same time, vast reservoirs of chemical diversity remain hidden within silent or cryptic biosynthetic gene clusters. Unlocking this latent potential stands as one of the most transformative future challenges. It will require activation strategies that merge ecological insight with chromatin biology, synthetic biology, and systems-level modeling [183,184]. As discussed in Section 7, the next generation of discovery efforts will likely rely on predictive frameworks that integrate genomics, ecology, and machine learning to anticipate when and where biosynthetic innovation is most likely to occur. Overall, natural product research is entering a phase defined not by incremental compound discovery but by conceptual expansion—a shift toward understanding natural chemistry as an adaptive, evolvable, and ecologically embedded system [185,186]. This integrated perspective not only enhances our capacity to discover novel metabolites but also deepens our appreciation of the biological strategies that shape life across kingdoms. Embracing this holistic view will be essential for unlocking the full biosynthetic potential of natural systems and for guiding future applications in medicine, agriculture, environmental science, and biotechnology.

## Figures and Tables

**Table 1:** Summary table of the principal classes of bioactive metabolites discussed in Section 5, highlighting their structural features, ecological functions, cross-kingdom distribution (plants, fungi, and marine organisms), biological activities, and associated reference numbers.

Aspect	Description	Biological/Chemical Relevance	Reference Numbers
Terpenoids	Structurally diverse metabolites from plants, fungi, and marine organisms involved in defense, communication, and stress adaptation.	Antimicrobial, cytotoxic, anti-inflammatory, and ecological signaling.	[62,63,64,65,66,67]
Polyphenols	Flavonoids, stilbenes, tannins, brominated and sulfated phenolics across kingdoms with roles in UV protection and stress resilience.	Antioxidant, anti-inflammatory, and neuroprotective properties.	[68,69,70]
Alkaloids	Nitrogen-containing metabolites from plants, fungi, and marine species mediate defense and inter-organismal communication.	Neuroactive, antimicrobial, cytotoxic, and cardiomodulatory effects.	[71,72,73,74]
Polyketides	Highly diverse metabolites produced mainly by fungi and marine organisms through modular biosynthesis.	Pharmacological relevance, including statins, activated via epigenetic modulation, co-culture, and CRISPR pathways.	[75,76,77,78,79,80]
Non-Ribosomal Peptides and Hybrids	NRPs, PKS–NRPS, and terpene–polyketide hybrids mediating ecological defense and competition.	Potent antimicrobial and anticancer properties; structurally innovative scaffolds.	[81,82,83,84]
Integrative Perspective	Chemical classes form a continuous biosynthetic landscape shaped by evolution and ecological pressures.	Reveals convergence of defense, signaling, and stress-response metabolites across kingdoms.	[85,86,87,88]

**Table 2:** Ecological interactions shaping the production of bioactive metabolites in plants and their symbiotic partners.

Ecological Interaction	Example Biological Systems	Induced/Enhanced Metabolites	Ecological–Chemical Mechanism	Relevance for *Plants*	Refs.
Plant–endophyte mutualism	Medicinal plants hosting endophytes (e.g., *Taxus*, *Artemisia*)	Terpenoids, alkaloids, phenylpropanoids	Co-regulation of BGCs; exchange of signaling molecules; shared defensive responses	Highlights plants as drivers of endophyte metabolic rewiring	[9,21,22]
Microbial competition in the rhizosphere	*Streptomyces*, rhizospheric fungi, PGPR bacteria	Natural antibiotics, siderophores, VOCs	Competition for nutrients and space; activation of silent gene clusters	Emphasizes plant-directed assembly of complex microbial networks	[18,19,20]
Fungal–fungal co-culture associated with plants	Endophytic Xylariales, *Aspergillus* spp. in interaction assays	Polyketides, NRPs, cryptic metabolites (up to 14× increase)	Antagonism and cross-kingdom signaling activate silent BGCs	Reveals plants as hotspots of ecologically driven chemical diversity	[24,25,26]
Abiotic stress (UV, salinity, drought)	Plants from extreme environments and their microbiomes	Flavonoids, brominated phenolics, protective terpenoids	Stress-induced defense pathways co-modulated by plant and symbionts	Shows how environmental pressures shape plant metabolomes	[68,69,70]
Plant–phytopathogen interactions	Powdery mildews, rusts, necrotrophic pathogens	Phytoalexins, oxidative metabolites, antimicrobial terpenoids	Rapid activation of defense pathways and specialized metabolism	Relevant for crop resilience and plant immunity	[99,105,107]
macrofungi/mushrooms and soil	AMF, ectomycorrhizal fungi	Sesquiterpenes, VOCs, organic acids	Hormonal modulation and nutrient exchange shape metabolite profiles	Connects soil biodiversity and plant chemical diversity	[29,31,32]

## Data Availability

No new data were created or analyzed in this study. Data sharing is not applicable to this article.

## Abbreviations

The following abbreviations are used in this manuscript:

AI	Artificial Intelligence
ABS	Access and Benefit-Sharing
ASCVD	Atherosclerotic Cardiovascular Disease
Bax/Bcl-2	B-cell lymphoma protein 2
BIOTA-FAPESP	FAPESP BIOTA Program—The Virtual Institute of Biodiversity
CBD	Convention on Biological Diversity
CBS	Corticobasal Syndrome
CRISPR/Cas9	Clustered Regularly Interspaced Short Palindromic Repeats
DNA	DeoxyriboNucleic Acid
DSI	Digital Sequence Information
EMA	European Medicines Agency
FDA	Food and Drug Administration
FUNGAL IDC	Fungal International Depository Center
GM	Genetically Modified
GMP	Good Manufacturing Practices
HMG-CoA	3-hydroxy-3-methylglutaryl coenzyme A
HRMS	High-Resolution Mass Spectrometry
ICH	International Council for Harmonisation of Technical Requirements for Pharmaceuticals for Human Use.
IPLCS	Indigenous Peoples and Local Communities
LC-MS/MS	Liquid Chromatography–Tandem Mass Spectrometry
NMR	Nuclear Magnetic Resonance
Nrf2-ARE	Nuclear factor erythroid 2–related factor 2—Antioxidant Response Element
NRPs	Non-Ribosomal Peptides
Nubbe	Nuclei of Bioassays, Biosynthesis and Ecophysiology of Natural Products
PAT	Process Analytical Technologies
Ppf4	Predator-Prey Factor at trophic level 4
PI3K/AKE/mTOR	Phosphoinositide 3-kinase/Protein kinase B (Akt)/Mechanistic target of rapamycin
RiPPs	Ribosomally Synthesized and post-translationally modified peptides
UKM	Universiti Kebangsaan Malaysia
VOCs	Volatile Organic Compounds
WHO	World Health Organization
